# Similarities and differences between helminth parasites and cancer cell lines in shaping human monocytes: Insights into parallel mechanisms of immune evasion

**DOI:** 10.1371/journal.pntd.0006404

**Published:** 2018-04-18

**Authors:** Prakash Babu Narasimhan, Leor Akabas, Sameha Tariq, Naureen Huda, Sasisekhar Bennuru, Helen Sabzevari, Robert Hofmeister, Thomas B. Nutman, Roshanak Tolouei Semnani

**Affiliations:** 1 Laboratory of Parasitic Diseases, National Institute of Allergy and Infectious Diseases, National Institutes of Health, Bethesda, MD, United States of America; 2 EMD Serono Research and Development Institute, Billerica, MA, United States of America; George Washington University, UNITED STATES

## Abstract

A number of features at the host-parasite interface are reminiscent of those that are also observed at the host-tumor interface. Both cancer cells and parasites establish a tissue microenvironment that allows for immune evasion and may reflect functional alterations of various innate cells. Here, we investigated how the phenotype and function of human monocytes is altered by exposure to cancer cell lines and if these functional and phenotypic alterations parallel those induced by exposure to helminth parasites. Thus, human monocytes were exposed to three different cancer cell lines (breast, ovarian, or glioblastoma) or to live microfilariae (mf) of *Brugia malayi–*a causative agent of lymphatic filariasis. After 2 days of co-culture, monocytes exposed to cancer cell lines showed markedly upregulated expression of M1-associated (TNF-α, IL-1β), M2-associated (CCL13, CD206), Mreg-associated (IL-10, TGF-β), and angiogenesis associated (MMP9, VEGF) genes. Similar to cancer cell lines, but less dramatically, mf altered the mRNA expression of IL-1β, CCL13, TGM2 and MMP9. When surface expression of the inhibitory ligands PDL1 and PDL2 was assessed, monocytes exposed to both cancer cell lines and to live mf significantly upregulated PDL1 and PDL2 expression. In contrast to exposure to mf, exposure to cancer cell lines increased the phagocytic ability of monocytes and reduced their ability to induce T cell proliferation and to expand Granzyme A^+^ CD8^+^ T cells. Our data suggest that despite the fact that helminth parasites and cancer cell lines are extraordinarily disparate, they share the ability to alter the phenotype of human monocytes.

## Author summary

Both cancer cells and parasites establish a tissue microenvironment that allows for immune evasion and may reflect functional alterations of various innate cells including monocytes. In recent years, there has been a large body of evidence in the field of cancer immunotherapy demonstrating that the immune system can be retrained to not only recognize tumor cells but also to eliminate them. Tumor immunology has advanced significantly with the advent of newer tools of molecular immunology and identification of tumor associated biomarkers. In fact, the concepts for immunotherapy as an effective way to reawaken the immune response against tumors that evade the immune system may apply to other diseases. Therefore, using similar approaches in infectious diseases, might lead to activation of the immune system and reversal of immunological ignorance against parasites. We strongly believe that a multidisciplinary approach would encourage crosstalk between different areas of science and will foster and advance research. Here, we investigate how cancer cell lines and helminth parasites shape the phenotype and function of human monocytes. Both cancer cell lines and helminth parasite altered the expression of selected genes associated with inflammatory, regulatory, or type 2 response and upregulated immune checkpoints PDL1 and PDL2 in human monocytes. Our data suggest that despite the fact that helminth parasites and cancer cell lines are different, they share the ability to alter the phenotype of monocytes.

## Introduction

A variety of mechanisms used by tumor cells to escape the host’s immune system are similar to those used by some parasites. Both parasites and tumors have developed strategies to escape the immune system by expanding T regulatory cells[1,2], by inducing the production of certain inhibitory cytokines[3,4], or by altering the function of antigen presenting cells (APCs) that, in turn, results in diminished ability of these cells to activate T cells[1,5,6].

Monocytes and macrophages are heterogeneous populations of cells that display high plasticity and are essential for the host innate immune response. Consequently, a change in their function contributes to alterations of immune function that may lead to dysregulation of responses important in limiting cancer progression[7] and constraining some infectious diseases. Based on responses to different stimuli, macrophages can be categorized into classically activated M1 (type 1 or pro-inflammatory activated by LPS or IFN-γ (Interferon-gamma)), or alternatively activated M2 (type-2 anti-inflammatory activated by IL-4 or IL-13)[8]. In fact, both parasites and tumors alter the balance of these monocyte /macrophage sub-populations[6,9–12].

When recruited to the tumor tissue, monocytes can differentiate into tumor associated macrophages (TAM), a heterogenous population of myeloid cells with both antitumor (M1) and pro-tumor activities (M2) (reviewed in[13]). In fact, TAMs have a wide range of functions including those with beneficial effects, such as phagocytosis of tumor cells and production of cytotoxic factors,[13,14] and the more deleterious effects, such as tumor- associated immunesuppression through the expression of inhibitory immune checkpoints PDL1 (CD274) and PDL2 (CD273)[15].

M2 macrophages with both anti-inflammatory and tissue repair functions[16,17], largely driven by IL-4 and/or IL-13 can also be induced by helminth parasites[6,18]. Helminth-induced M2 macrophages have been shown to play a role in control of Th1-type inflammation, worm expulsion, and wound healing in murine models[19]. Interestingly, microfilariae (mf) of *Brugia malayi*, the bloodborne stage of one of the helminth parasites that cause lymphatic filariasis in humans, alter monocyte populations somewhat differently in that both M1 and M2 phenotypes are induced[6,18,20,21]. Furthermore, monocyte dysfunction[20,22] of either subset in filarial infection is one of the many mechanisms proposed for parasite antigen-specific T cell hyporesponsiveness seen in humans with lymphatic filariasis.

Because the regulation of monocyte function plays a critical role in both helminth infection and tumor progression, in the present study we assessed the similarities and differences between parasite and cancer-induced alterations of both phenotype and function of human monocytes in hopes of identifying potential new targets that can be exploited by host directed therapeutics. Breast and ovarian cancers are considered to be among the leading types of cancer in North America and Europe [23,24]. Clinical data demonstrate a strong relationship between increased monocyte/macrophage density and poor prognosis in breast and/or ovarian cancers and glioblastoma [25,26]. In addition, tumor associated macrophages play an important role in all three cancer types [27–29]. Therefore, in the present study we chose breast, ovarian, and glioblastoma cancer cell lines to compare their effects on human monocytes to that of helminth parasites.

## Methods

### Ethics statement

The elutriated monocytes and lymphocytes from leukopacks of healthy adult donors from North America were collected by counterflow centrifugal elutriation under a protocol approved by the Institutional Review Board (IRB) of the Department of Transfusion Medicine, Clinical Center, National Institutes of Health (NIH; IRB 99-CC-0168). The healthy adult volunteers were given informed written consent.

### mf preparations

Live *Brugia malayi* mf (provided under contract with the University of Georgia, Athens, GA) were collected by peritoneal lavage of infected jirds and separated from peritoneal cells by Ficoll diatrizoate density centrifugation. The mf were then washed repeatedly in RPMI medium with antibiotics and cultured overnight at 37°C in 5% CO2 before use.

### Cell lines

The breast cancer; MDA-MB-231 (MDA), ovarian cancer; OVCAR-3 (OVCAR), and glioblastoma; U87-MG (U87) cell lines were obtained from American Type Culture Collection (ATCC) (Manassas, VA). MDA cells were cultured in Dulbecco’s Modified Eagle’s Medium (DMEM, ATCC 30–2002) containing 10% heat–inactivated fetal bovine serum (Gemini Bioproducts, Sacramento, CA). OVCAR cells were cultured in Roswell park memorial institute medium (RPMI-1640, ATCC 30–2001) containing 20% heat–inactivated fetal bovine serum. U87 cells were cultured in Eagle’s Minimum Essential Medium (EMEM, ATCC 30–2003) containing 10% heat–inactivated fetal bovine serum. All culture media contained 100 Units/ml penicillin and 0.1-mg/ml streptomycin ([P/S] Biofluids, Inc, Rockville, MD). Cells were cultured at 37° C in humidified air at 5% CO_2_ and were confirmed to be devoid of mycoplasma. Prior to use, these cell lines were stained with 0.5uM, 5-chloromethylfluorescein diacetate (Cell Tracker green CMFDA; Molecular probes) in serum free media for 30 min followed by washing with PBS.

### *In vitro* exposure of monocytes to cancer cell lines and live mf

CMFDA labeled cancer cell lines (MDA, OVCAR and U87) were cultured for 24 hours prior to co-culture with monocytes. Human monocytes were cultured at 50 × 10^6^ per 6-well plate in serum-free media RPMI 1640 medium supplemented with 20 mM glutamine (Lonza) P/S for 2 h, after which the medium was removed and the adherent cells were harvested. Monocytes were then either cultured alone or exposed to live mf (50,000 per million cells to reflect physiologically relevant concentrations), or to three different CMFDA labeled cancer cell lines at a 1:2 (cancer cell lines: monocytes) ratio for 48hrs. For monocyte co-cultures with each cancer cell lines, we chose the media used for culturing the relevant cancer cell line alone. Therefore, for MDA cell line and the co-culture of monocytes and MDA (mon/MDA) DMEM complete media; for OVCAR and co-culture of monocytes and OVCAR (mon/OVCAR) RPMI complete media; and for U87 and co-culture of monocytes and U87 (mon/U87) EMEM complete media was used. Furthermore, monocytes alone or monocytes exposed to mf were cultured in DMEM complete media as there was no difference between the three-different media in mRNA expression, cell surface expression or viability of monocytes (S1 Fig).

After 48hrs, the cells were harvested by cell scraping (Corning Costar) and washed once with PBS (without Ca^++^/Mg^++^), counted monocytes were first incubated with human gammaglobulin (Sigma) at 10 mg/ml for 10 min at 4°C to inhibit binding of the monoclonal antibody to Fc receptor (FcR) and were subsequently labeled with mouse phycoerythrin labeled anti-CD45 mAb (eBioscience, San José, CA; Cat No. 12-9459-42), at saturating concentrations for 30 min at 4°C. The cells were then washed twice with FACS medium and sorted on FACSAria III, 6-laser, 15-parameter, cell sorter (Becton Dickinson, Sparks, MD) by gating on the expression of CD45 and lack of CMFDA (CD45^+^/CMFDA^-^). Sorted monocytes were then used for gene expression or functional analysis.

### Cytokine measurements

After 48hrs of monocytes co-culture with cancer cell lines or mf, exposed and unexposed sorted monocytes were cultured in DMEM media overnight without any stimulation and the production of TNF-α (Tumor necrosis factor-alpha), IP-10 (Interferon gamma-induced protein (CXCL10)), IL-6, CCL4 (Macrophage inflammatory protein 1-β (MIP-1β)) and CCL22 (macrophage derived chemokine (MDC)) in the culture supernatants were measured using a Multiplex human cytokine/chemokine magnetic bead panel kit (EMD Millipore, Billerica, MA) and a Luminex 100/200 system (Luminex, Austin, TX). The lower limit for detection for these assays was 3.2 pg/ml.

### Phagocytosis

Phagocytic activity of the human monocytes was assessed by a phagocytosis assay kit (Molecular probes; Invitrogen) with some modifications. Briefly, sorted monocytes (1.0 X10^6^ cells/well) were incubated with 10^8^ inactivated *E*.*coli* Alexa 488 Bioparticles for 1 hr at 37° C, 5% CO_2_ in serum free DMEM media. The cells were washed with PBS, incubated for 1 min with 0.4% trypan blue to quench any extracellular fluorescence, and washed twice with PBS. Intracellular fluorescence intensity was quantified by flow cytometry. All experiments were done with six replicates. Phagocytic activities of monocytes were expressed as percent phagocytosis relative to that seen with controls.

### Flow cytometry staining

Human monocytes were cultured alone or with mf or three different CMFDA-labeled cancer cell lines (green; FITC channel) as mentioned above. After 48 hrs, cells were harvested, washed with PBS and incubated with 10 μl human IgG (10 mg/ml; Sigma-Aldrich, St. Louis, MO) for 10 min at 4°C to inhibit nonspecific binding through FcγRs and then incubated with marker-specific mAb conjugated with PDL2- APC (eBioscience, Cat No. 17-5888-42), VCAM1(Vascular cell adhesion molecule-1)-APC (Biolegend, Cat No. 305810), CD14-APC Cy7 (eBioscience, Cat No. 47-0149-42), CD45-Pacific blue (Biolegend, Cat No. 304022), CD206 (Mannose receptor)-Percp efluor 710 (eBioscience, Cat No. 46-2069-42), PDL1-PE-Cy7 or PDL1-APC (eBioscience, Cat No. 25-5983-42 and Cat No. 17-5983-41 respectively), or CD163-PE (eBioscience, Cat No. 12-1639-42), CD45-PE (eBioscience, Cat No. 12-9459-42) at saturating concentrations for 30 min at 4°C and washed twice with FACS medium. Monocyte cell populations (CD45^+^/CMFDA^-^) were then identified and gated to measure the expression of cell surface markers.

### Flow cytometric analysis

For flow cytometric analysis, 50,000 events were acquired per tube using a BD LSRII flow cytometer (BD Biosciences, San Jose, CA). Compensation was performed in every experiment using BD CompBeads (BD Biosciences) for single-color controls and unstained cells as negative controls. Data were analyzed using FlowJo Software (Tree Star, Ashland, OR). Nonviable cells were excluded from our analysis on the basis of forward and side scatter. Fold upregulation in Mean Fluorescence Intensity (MFI) was measured for all markers.

### RNA preparation and real-time RT-PCR

Exposed and unexposed sorted monocytes were used for isolation of total RNA and RT-PCR to measure gene expression. Total RNA was prepared from 8 to 15 independent donors using an RNAEasy minikit (Qiagen). RNA (1 μg) from the cells was used to generate cDNA and then assessed by standard TaqMan assays (Applied Biosystems Inc.) using an ABI 7900HT system (Applied Biosystems, Inc.). Briefly, random hexamers were used to prime RNA samples for reverse transcription using MultiScribe reverse transcriptase (Applied Biosystems Inc.), after which PCR products for all genes, as well as an endogenous 18s rRNA control, were assessed in triplicate or duplicate wells using TaqMan predeveloped assay reagents. The threshold cycle (CT), defined as the PCR cycle at which a statistically significant increase in reaction concentration is first detected, was calculated for the genes of interest and the 18S control and used to determine relative transcript levels.

Relative transcript levels were determined by the formula 1/ΔCT, where ΔCT is the difference between the CT of the target gene and that of the corresponding endogenous 18S reference. Fold change in gene expression was measured using 2^−−ΔΔCT^, where ΔΔCT is the difference between the ΔCT of the gene of interest in exposed monocytes and that of the unexposed control.

### T cell proliferation and Granzyme A expression

Exposed or unexposed sorted monocytes were cultured with CellTrace carboxyfluorescein succinimidyl ester (CFSE) (ThermoFisher Scientific, Cat No. C34570)- autologous and allogeneic lymphocytes (1:1 monocyte/T cell ratio) either in media alone or with 10ug/ml of anti-CD3 (hOKT3) in 24 well tissue culture plates (Costar, Cambridge, MA). Blocking experiments were done using anti-PDL1 (eBiosciences, Cat No. 16-5983-82) and isotype control IgG1 (eBiosciences, Cat No. 16–4714) at a final concentration of 10ug/ml. After 4 days of culture, CFSE-labeled lymphocytes were harvested, and the proliferation of CD4^+^ and CD8^+^ T cells was measured by flow cytometry using a combination of APC conjugated anti-CD4 (BD Biosciences, Cat No. 340672) and Alexa Fluor 700-conjugated anti-CD8 (eBiosciences, Cat No. 56-0086-42). The cells were then analyzed by acquisition of 50,000 events/tube using a BD LSRII (BD Biosciences). Compensation was performed in every experiment using BD CompBeads (BD Bioscience) for single-color controls and unstained cells. Nonviable cells were excluded from the analysis based on forward and side scatter. CellTrace Violet labeled lymphocytes were further gated on expression of CD4^+^ or CD8^+^, and proliferation was measured by flow cytometry for the dilution of fluorescent dye. Proliferation indices were calculated by using the FlowJo proliferation analysis program (Tree Star, Ashland, OR). Granzyme A expression was measured in CD8^+^ T cells by staining with pacific blue anti-granzyme A antibody (Biolegend, Cat No. 515407).

### Statistical analysis

Unless noted otherwise, geometric means were used as a measure for central tendency. Omnibus2 normality test was performed to confirm the data is not normally distributed and then the nonparametric Wilcoxon signed-rank test was used for paired group comparisons. All analyses were performed using GraphPad Prism 6.0 (GraphPad Software, Inc., San Diego, CA).

## Results

### mRNA expression of selected genes associated with inflammation, type 2, regulatory and angiogenesis in human monocytes after exposure to either cancer cell lines or mf

**Fig 1:**
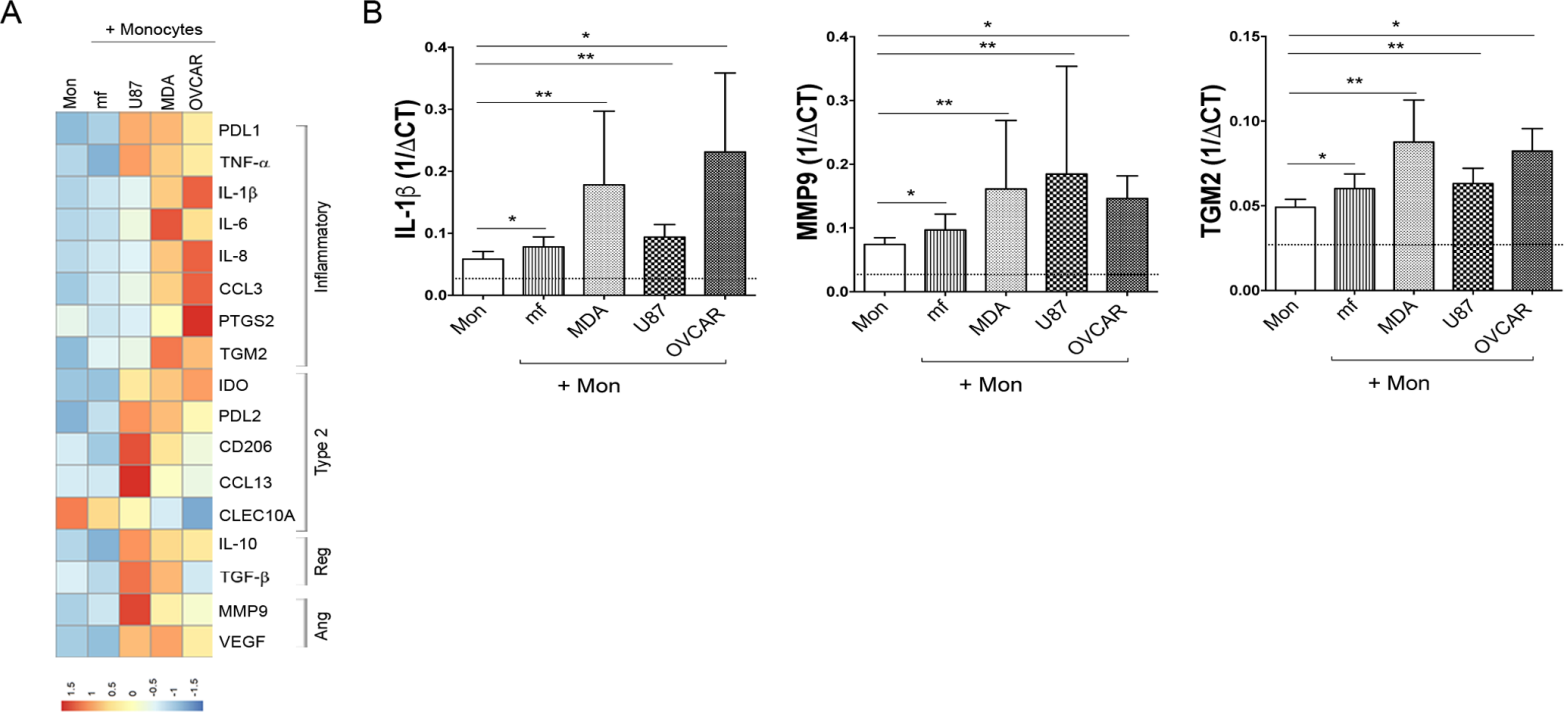
mRNA expression of selected genes associated with inflammation, type 2, regulatory, and angiogenesis. Human monocytes were either unexposed (Mon) or exposed to CMFDA-labeled three different cancer cell lines (MDA, OVCAR, U87), or to live mf of *Brugia malayi* for 48 hours. CD45^+^/CMFDA^-^ monocytes were sorted and mRNA levels were measured by TaqMan real-time PCR and normalized to the levels of 18S rRNA. A) Heat map of differential expression of selected inflammatory, type-2, regulatory (Reg) and angiogenesis (Ang) related genes (geometric means of 1/delta CT; n = 10). The intensity of blue to red denotes the low to high expression of genes respectively. B) mRNA expression of IL-1β, MMP-9 and TGM2. The data are expressed as the geometric mean with 95% confidence interval of 1/delta CT (n = 10). * *P*<0.05, ** *P*<0.005.

To assess whether cancer cell lines and helminth parasites share similar features in shaping human monocyte gene expression, human monocytes were either exposed to CMFDA- labeled cancer cell lines (breast cancer; MDA-MB-231 (MDA), ovarian cancer; OVCAR-3 (OVCAR), and glioblastoma; U87-MG (U87)) or to live mf of *Brugia malayi* for 48 hours, sorted for CD45^+^/CMFDA^-^ monocytes and assessed for mRNA expression by RT-PCR. To further assess the phenotype of exposed monocytes, we selected genes associated with inflammatory response (M1), type-2 response (type 2/M2), regulatory response (Reg) or responses associated with angiogenesis (Ang) (Fig 1A and 1B and S2 Fig). Our results indicate that cancer cell lines significantly upregulated genes associated with M1 monocytes including PDL1, TNF-α, IL-1β, IL-6, IL-8, CCL3, and prostaglandin-endoperoxidase synthase 2 (PTGS2) (Fig 1A and S2 Fig). The expression of genes associated with M2 monocytes (TGM2 (Transglutaminase 2), PDL2, CD206, CCL13), regulatory monocytes (IL-10, TGF-β) and angiogenesis (MMP9 (Matrix metallopeptidase-9), and VEGF (Vascular endothelial growth factor)) were significantly induced in all three different cancer cell line- exposed monocytes when compared to unexposed monocytes (Fig 1A and S2 Fig). Although there were major differences between cancer cell line exposed- and mf-exposed monocytes, mRNA expression of IL-1β, MMP9, and TGM2 was shown to be significantly upregulated in monocytes following exposure to either stimuli compared to unexposed monocytes (Fig 1B).

### Cancer cell lines and mf significantly upregulate the production of IP10 and CCL22 in human monocytes

**Fig 2:**
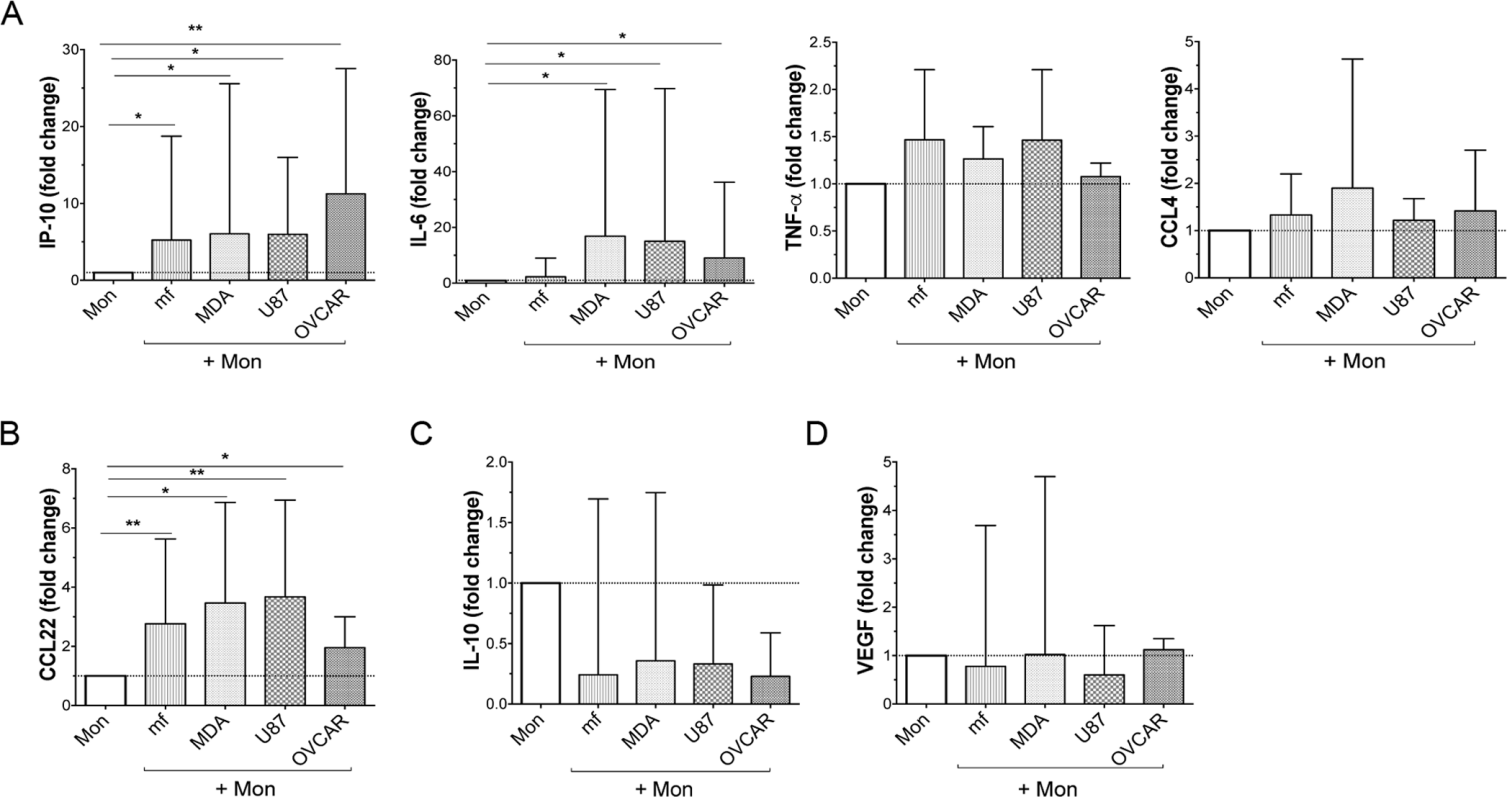
Cancer cell lines and mf significantly upregulate the production of IP-10 and CCL22 in human monocytes. Human monocytes were cultured in media alone (Mon), or with CMFDA-labelled three different cancer cell lines (MDA, OVCAR, U87), or with live mf of *Brugia malayi* for 48hr. CD45^+^ /CMFDA^-^ monocytes were then sorted and cultured in media alone. Supernatants were collected after an additional 24 h and evaluated for levels of A) inflammatory, B) Type 2, C) regulatory, or D) cytokines related to angiogenesis by Luminex. The data are expressed as the geometric mean with 95% confidence interval of the fold change over unexposed monocytes (*n* = 5–12). * *P*< 0.05, **P<0.005.

We next measured monocyte cytokine production (Fig 2) after exposure to cancer cell lines or mf. To further assess the phenotype of exposed monocytes, we selected cytokines associated with inflammatory response (M1), type-2 response (type 2/M2), regulatory response or responses associated with angiogenesis (Fig 2 and S1 Table). To this end, human monocytes were either exposed to CMFDA- labeled cancer cell lines (MDA, OVCAR, and U87) or to live mf of *Brugia malayi* for 48 hours, sorted and rested in media for an additional 24 hours following which cytokine production was assessed in the culture supernatant. As seen in Fig 2, both cancer cell lines and live mf significantly (p = 0.001) upregulated the production of IP-10 and CCL22, while IL-6 production was significantly upregulated only by cancer cell lines and not by mf (Fig 2). Among cytokines/chemokines tested the production of TNF-α, CCL4 (Fig 2A), IL-10 (Fig 2C), and VEGF (Fig 2D) was not affected by exposure to any of the stimuli.

### Both mf and cancer cell lines induce the monocyte cell surface expression of PDL1, PDL2, VCAM-1, and CD206

**Fig 3:**
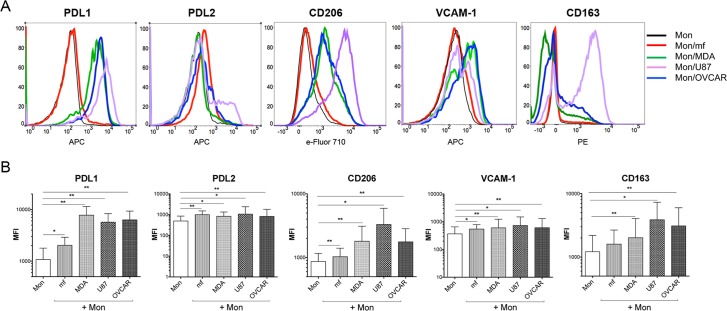
Cancer cell lines and mf significantly upregulates the cell surface expressions of PDL1, PDL2, CD206 and VCAM-1 on human monocytes. Human monocytes were cultured in media alone, or with CMFDA-labeled three different cancer cell lines (MDA, OVCAR, U87), or with live mf of *Brugia malayi* for 48hr. Cells were harvested and cell surface expression PDL1, PDL2, CD206, VCAM-1, and CD163 was measured using flow cytometry gated on CD45^+^/CMFDA^-^ monocytes. (A) One representative set (n = 15) of flow histograms demonstrating cell surface expression in unexposed human monocytes and after exposure to mf or different cancer cell lines. (B). The data are expressed as the geometric mean with 95% confidence interval of the mean fluorescent intensity of unexposed and exposed monocytes (*n* = 15). * *P<* 0.05, ** *P<*0.005.

We then determined the phenotype of the monocytes following exposure to either live mf or to the cancer cell lines. The basal expression of selected cell surface markers (M1, M2, inhibitory) on either human monocytes or cancer cell lines suggest that human monocytes do not express PDL2 (M2/inhibitory), VCAM-1 (M1), CD206 (M2), and have a low expression of PDL1 (M1/inhibitory) and CD163 (M2) (Fig 3A, black lines). To our interest, after 48 hours exposure to either of the three cancer cell lines, the expressions of PDL1 (p = 0.001), PDL2 (p = 0.003), VCAM-1 (p = 0.003), and CD206 (p = 0.003) on monocytes were significantly upregulated (Fig 3A and 3B). Similar to cancer cell lines, but to a lesser extent, live mf significantly upregulated the cell surface expression of each of these markers (p = 0.001) with the exception of CD163 (Fig 3A and 3B). Longer exposure (5 days) of monocytes to mf further induced the level of cell surface PDL1 (S3A and S3B Fig).

### Cancer cell lines and MCSF (but not mf) significantly induce the phagocytic ability of human monocytes

**Fig 4:**
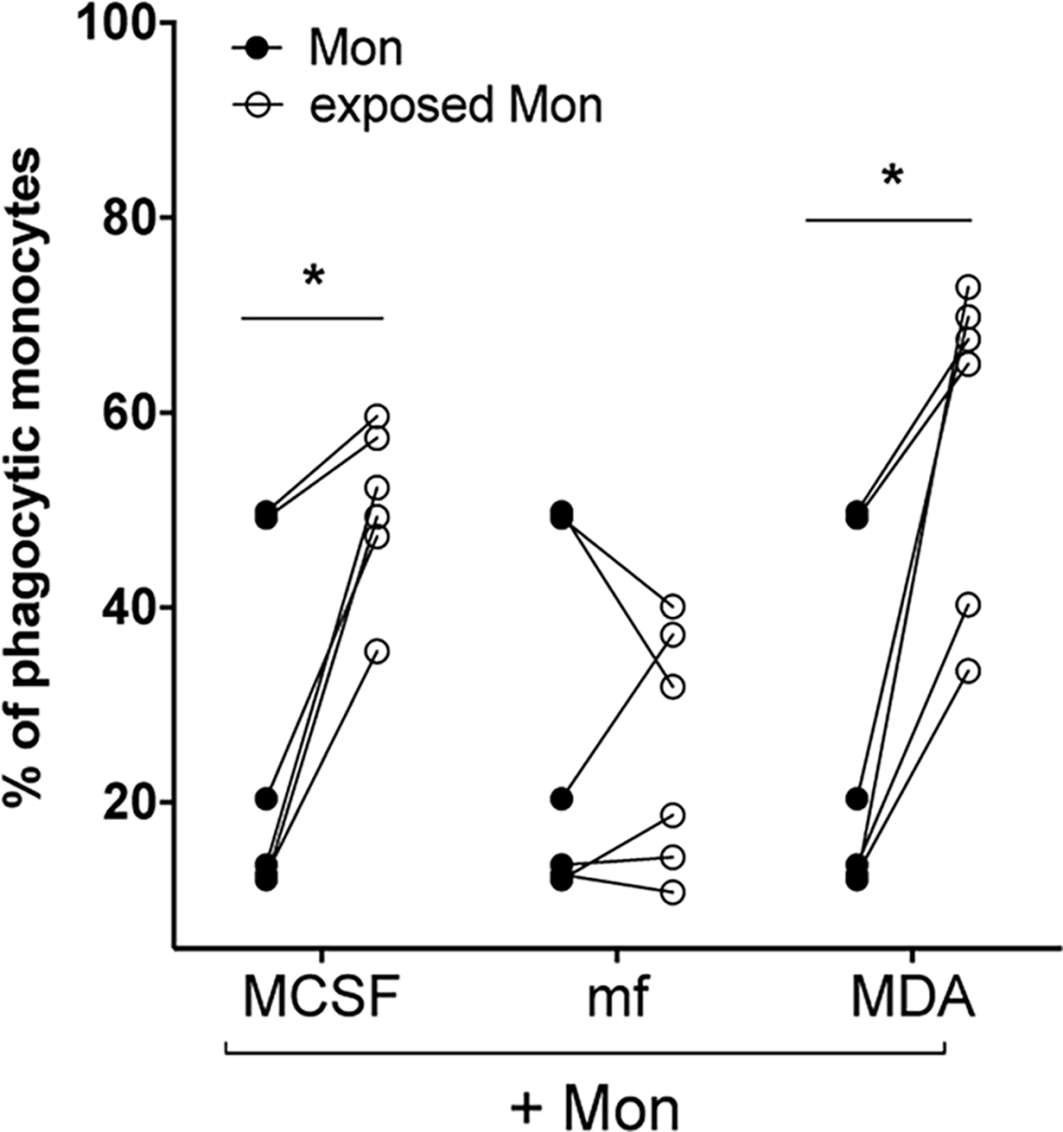
Breast cancer cell line MDA and MCSF (but not mf) significantly induce the phagocytic ability of human monocytes. Human monocytes were cultured in media alone, or with CMFDA-labeled MDA, or live mf of *Brugia malayi* for 48hr. Cells were harvested and CD45^+^/CMFDA^-^ monocytes were sorted and cultured to measure phagocytosis of opsonized fluorescent- labeled *E*. coli bioparticles (see Materials and Methods). Results are shown as the percentage of phagocytic monocytes (FITC labeled *E*. *coli* positive). Each circle represents an independent donor, where closed circles represent unexposed monocytes and open circles represent monocytes exposed to either MCSF, MDA, or live mf. * *P* < 0.05.

To compare the ability of cancer cell lines and mf to shape the phagocytic function of human monocytes, we measured the ability of monocytes to take up fluorescently labeled *E*.*coli* following exposure to mf, or MDA (Fig 4), or OVCAR and U87 (S4 Fig). Our data clearly show that similar to MCSF-treated monocytes, exposure to MDA (Fig 4, P = 0.01) or to the other two cancer cell lines (S4 Fig) significantly induce the phagocytic ability of these cells. However, live mf did not alter the ability of monocytes to phagocytose *E*. *coli* bioparticles (Fig 4).

### Breast cancer cell line MDA significantly inhibit the proliferation of allogeneic and autologous CD4^+^ T cells in a PDL1-dependent manner and reduce the frequency of Granzyme A^+^ CD8^+^ T cells

**Fig 5:**
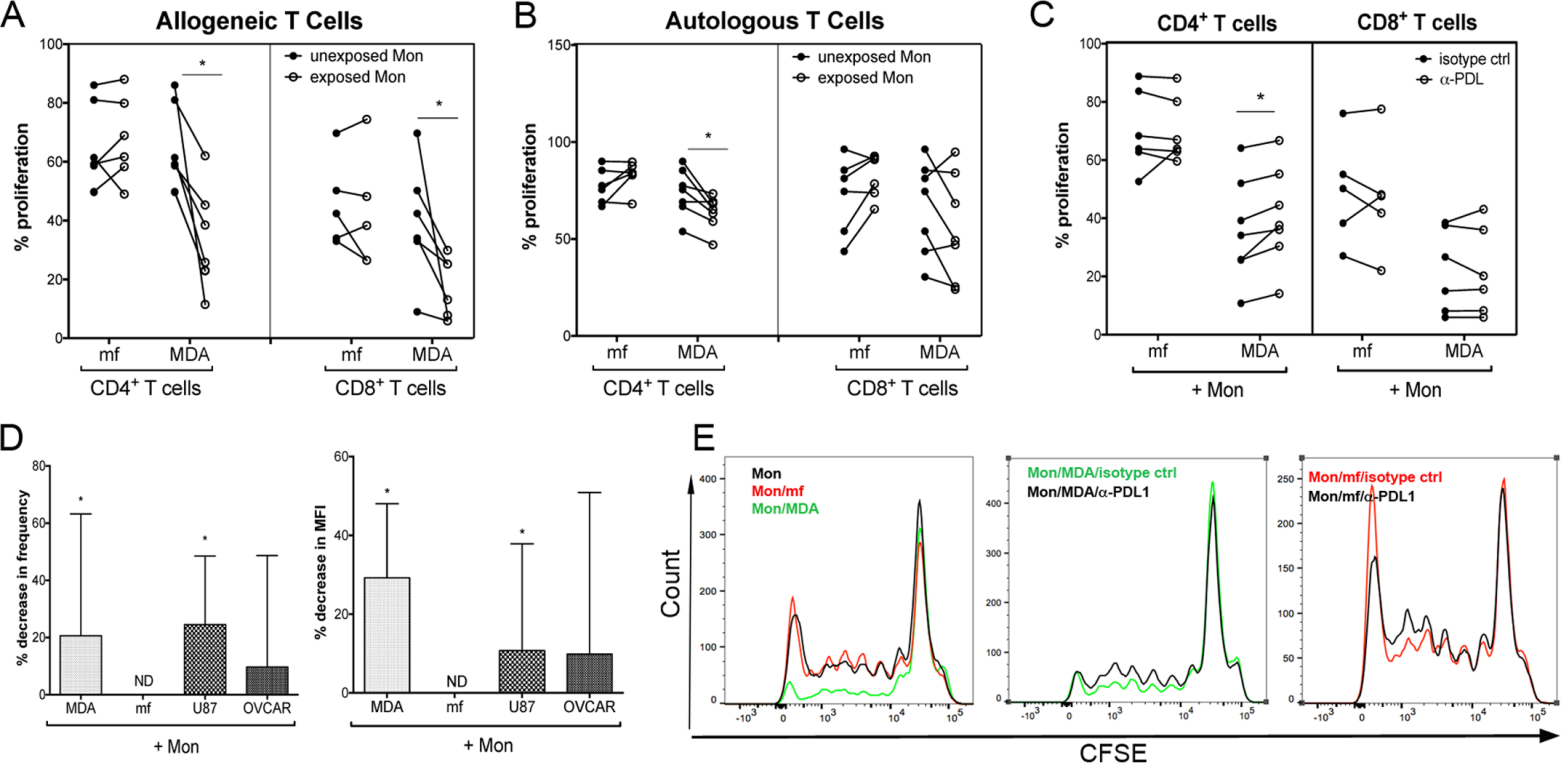
Cancer cell lines significantly diminishes PDL1-dependent proliferation of allogeneic and autologous CD4^+^ T cells and significantly diminishes the frequency of Granzyme A^+^ CD8^+^ T cells. Human monocytes were cultured in media alone, or with CMFDA-labeled MDA-MDA or live mf of *Brugia malayi*, for 48hr. Cells were harvested and CD45^+^/CMFDA^-^ monocytes were sorted and co-cultured with CFSE-labeled A) allogeneic or B) autologous lymphocytes in the presence of soluble anti-CD3 (10ug/ml) for an additional 4 days. Percent proliferation of CD4^+^ and CD8^+^ T cells was measured by flow cytometry (n = 7) either A and B) in the absence of antibody or C) in the presence of isotype control (closed circle) or anti-PDL1 (open circle). Each line represents an independent donor. *, *P* < 0.05. D) Frequency and MFI of allogeneic Granzyme A^+^ CD8^+^ T cells was measured by flow cytometry. The data are expressed as geometric mean of percent decrease in frequency and MFI of Granzyme A^+^/CD8^+^ T cells. ND = No Decrease; *P < 0.05. E) One representative set (n = 7) of flow histograms demonstrating proliferation of allogeneic CD4^+^ T cells either without antibody (first panel), or in the presence of isotype control or α-PDL1 (second and third panels).

The suppressive effects of tumor-associated macrophages on T cell proliferation have been shown previously[30]. To assess whether these particular cancer cell lines or live mf alter the ability of monocytes to drive T cell proliferation and mediator release, human monocytes were cultured in media alone, or with CMFDA-labeled breast cancer cell lines or live mf of *Brugia malayi* for 48hr. CD45^+^/CMFDA^-^ sorted exposed and unexposed monocytes were then co-cultured with CFSE-labeled allogeneic or autologous lymphocytes in the presence of anti-CD3 for an additional 4 days. While exposure to mf did not alter the ability of monocytes to induce T cell proliferation, exposure to MDA and the other two cancer cell lines (U87 and OVCAR; S5 Fig) significantly diminished their ability to promote allogeneic and autologous CD4^+^ (Fig 5A and 5B), and allogeneic CD8^+^ (Fig 5A) T cell proliferation.

We next aimed to investigate the role of PDL1 in this diminished T cell proliferative activity. As shown in Fig 5C and 5E, blocking the PDL1 pathway significantly increased the proliferation of allogeneic CD4^+^ T cells, suggesting that the PDL1 upregulation in cancer cell line exposed- (but not mf-exposed) monocytes plays an important role in T cell suppression.

Because regulation of cytolytic CD8^+^ T cells is crucial in controlling tumor progression and growth particularly through the release of granzymes (reviewed in[31]), we studied the effect of cancer cell line exposed monocytes on granzyme A release in CD8^+^ T cells. As shown in Fig 5D, MDA- and U87- exposed monocytes but not OVCAR- or mf-exposed monocytes significantly decreased the percentage of Granzyme A^+^ allogeneic CD8^+^ T cells when compared to the unexposed monocytes. Furthermore, longer exposure of monocytes to mf (5 days) did not result in a decrease in allogeneic or autologous (α-CD3 dependent) CD4^+^ or CD8^+^ T cell proliferation (S6A and S6B Fig). As well, the further increase in cell surface expression of PDL1 on monocytes after 5 days exposure to mf (S3 Fig) did not result in inhibition of T cell proliferation (S6A and S6B Fig).

## Discussion

Within most solid tumors, monocytes and macrophages are the major inflammatory infiltrates that can be recruited to the tumor microenvironment by tumor-derived chemokines, cytokines and other signals[32]. The majority of these infiltrating cells differentiate into TAMs promoting cancer cell proliferation, immunosuppression, and angiogenesis[33–35]. While the immunosuppressive TAMs are activated by IL-4 and IL-13 (Th2-associated cytokines), IL-10, glucocorticoids and vitamin D3, and can exert functions similar to M2 macrophages[36], recent findings suggest that some TAMs are Th1-skewed and resemble M1 macrophages[37]. In murine models, glioblastoma-associated monocytes/macrophages have shown to produce a broad range of cytokines/chemokines with both anti- and pro-inflammatory properties[38]. However, in humans, the same monocytes have phenotypes that are largely anti-inflammatory[39]. In addition, monocytes co-cultured with breast cancer cell lines have shown to have pro-tumor patterns of activities[40]. Therefore, TAMs are not simply restricted to M1 and M2 phenotypes and can represent a spectrum depending on tumor type, location, and microenvironment (reviewed in[41]).

In general, helminth parasites are shown to induce an M2 response in monocyte/macrophage populations (reviewed in[42]). Although, it has already been reported that both helminths and tumors alter the function of monocytes/macrophages, there are no studies assessing the similarities and differences between parasites and cancer cells in their effect on the phenotype and function of these cells. Given that both parasites and tumors share features designed to manipulate the host immune response, we aimed to study the similarities and differences between them with respect to monocytes (S1 Table).

To do so, we established a comparison between three cancer cell lines (MDA, OVCAR, and U87) and the circulating stage of the helminth parasite, *Brugia malayi* and then assessed the phenotype and function of human monocytes after exposure to either cancer cell lines or parasites. While we have looked at gene expression and cytokine production of mf- and cancer cell lines-exposed monocytes at various time points (5 or 7 days; S7 Fig), we chose 48 hours as mf exert its profound effect on DC and monocytes at this time point[6,9,43,44].

In our hands, and in agreement with previous studies[45,46] upon exposure of monocytes to cancer cell lines, both pro- and anti-inflammatory genes are induced (Fig 1, S2 Fig and S1 Table). In fact, all three-cancer cell lines significantly enhanced the monocyte mRNA expression of genes associated with inflammatory responses, Type-2 responses, regulatory responses, and angiogenesis (Fig 1 and S2 Fig). The M2 differentiation of monocytes co-cultured with breast cancer cell lines in transwell has been shown in the past[46]. In our studies, co-culture of monocytes with the supernatant of cancer cell lines resulted in enhanced expression and frequency of CD206 and PDL1 positive cells (S8 Fig).

Furthermore, cancer cell lines- exposed human monocytes demonstrated a significant induction of TGF-β, PGE2 and IL-10 as compared to unexposed cells (Fig 1). These immunosuppressive cytokines are known to be produced by macrophages in the tumor microenvironment to promote tumor growth by maintaining T regulatory cell differentiation[11,47]. Moreover, angiogenesis is a key event in tumor growth and progression, and TAMs are the major cell types promoting this event by producing factors such as VEGF in the tumor microenvironment[48,49]. For example, the interaction between monocytes/macrophages and ovarian cancer cells results in an increased ability of endothelial cells to promote tumor progression through angiogenesis[33]. Here our data indicate that exposure of human monocytes to all three-cancer cell lines results in significant induction of VEGF and MMP9 (Fig 1) suggesting a phenotype similar to TAMs.

One of the major similarities between mf- and cancer cell line- exposed monocytes is the significant upregulation in the mRNA levels of IL-1β (associated with inflammation or M1 phenotype), MMP9 (associated with angiogenesis), and TGM2 (associated with M2 phenotype) (Fig 1B). However, the magnitude of this upregulation is less profound in mf-exposed monocytes (Fig 1A and 1B).

Another similarity between cancer cell lines and mf is in their regulation of cytokine production. For example, while neither (mf or cancer cell lines) induces the production of CCL4, TNF-α, IL-10, or VEGF, they both significantly enhance the production of IP-10 and CCL22 in human monocytes (Fig 2). Both IP-10 and CCL22 are involved in lymphocyte chemotaxis and recruiting regulatory T cells to the tumor microenvironment[50,51]. IP-10 also binds endothelial cells and exerts a potent angiogenic activity in tumor settings[52]. While the role of IP-10 in T cell recruitment has been shown with intracellular parasites[53], the importance of this chemokine in helminth infection is still not fully understood. In humans, CCL22 and CCL18 are the chemokines expressed by M2 macrophage[54,55] and mf of *Brugia malayi* upregulate the mRNA expression of both chemokines[6] and also induce the production of CCL22 (Fig 2).

An important similarity between helminth parasites and cancer cell lines demonstrated here is their ability to upregulate monocyte cell surface expression of inhibitory molecules such as PDL1 and PDL2 (Fig 3). While unexposed monocytes have low expression of PDL1, CD163, and CD206 (Fig 3A; black solid lines), exposure to mf, similar to those of cancer cell lines, significantly upregulate the cell surface expression of PDL1 (inhibitory), PDL2 (M2/inhibitory), CD206 (M2), and VCAM-1(M1) (Fig 3A and 3B). Interestingly, while the upregulation in cell surface expression of PDL1 on monocytes was less profound with mf than with cancer cell lines, longer exposure to this parasite further increased the level of PDL1 (S3 Fig).

PDL1 and PDL2 are the two ligands for a major immune-checkpoint receptor PD1 (CD279)[56,57]. The engagement of PD1 on T cells with its ligands (PDL1/PDL2) on APCs inhibits kinases that are involved in T cell activation[56,58]. The majority of lymphocytes that infiltrate tumor microenvironment express PD1 and acquire a phenotype of hyporesponsiveness[59,60]. On the other hand, PDL1 is shown to be on most melanoma, ovarian and many other cancer types[61]. In addition to tumors, myeloid cells in tumor microenvironment such as TAMs also express high levels of PDL1 and PDL2[62–67]. Therefore, blockade of this inhibitory pathway is essential in cancer immunotherapy (reviewed in[62]).

Our data suggest that monocytes that are exposed to MDA (Fig 5; and other cancer cell lines, S5 Fig) have significantly decreased their ability to promote allogeneic CD4^+^ and CD8^+^ and autologous CD4^+^ T cell proliferation as compared to unexposed monocytes (Fig 5A and 5B), suggesting a suppressive phenotype. Interestingly, blocking PD1/PDL1 pathway with anti-PDL1 mAb reversed the suppressed proliferation in allogenic CD4^+^ but not CD8^+^ T cells that were co-cultured with MDA exposed monocytes (Fig 5C).

Similar to cancer settings, chronic filarial infection with continuous release of parasite antigens is associated with a lack of CD4^+^ T cell proliferation and production of IFN-γ and IL-2[68]. The role of PD1/PDL1 (PDL2) in regulating T cell response has also been extended to several infections[69]. Recent studies have suggested that macrophage expression of PDL1 is important in regulating T cell responses to influenza infection[70]. In acute malaria the induction of PD1^+^CTLA4^+^ effector T cells results in suppressive function and inhibition of other CD4^+^ T cells[69]. In our study, one major difference between helminth parasites and cancer cells is how they shape monocytes to promote T cell activation (Fig 5). In contrast to cancer cells, exposure to mf did not diminish the ability of monocytes to promote T cell proliferation (Fig 5A, 5B and 5C). Therefore, blocking PDL1 pathway in mf-exposed monocytes did not have any effect on T cell proliferation. Furthermore, longer exposure of monocytes to mf (5 days, S6 Fig) does not inhibit T cell proliferation. While, it has been suggested that other inhibitory molecules such as CTLA4 can play a role in T cell hyporesponsiveness seen in filarial-infected individuals[71], how PD1/PDL1 may play a role in this suppression is not known.

The ability of cytotoxic lymphocytes to recognize and kill infected or transformed cells is an important part of both innate and adaptive arms of the immune system. In fact cytolytic T lymphocytes are key players in current immunotherapies and promote apoptosis of cancer cells through granule-mediated as well as receptor-mediated mechanisms (reviewed in[31]). Stimulation of these cytolytic T cells through their receptors induces the activation of effector mechanisms including the granule exocytosis pathway releasing granule-associated enzymes (granzymes) as well as other factors resulting in target cell death[72]. Granzyme A and B are two important serine proteases that are involved in lymphocyte mediated cytotoxicity[31]. In fact, it has been shown that inhibition in the function of these cytolytic T lymphocytes such as CD8^+^ T cells by TAM establishes a suppressive microenvironment for the infiltrating immune cells[73,74].

Here, we demonstrate that exposure of human monocytes to MDA and U87 cancer cells, but not to OVCAR or mf (Fig 5D) significantly downregulated the percentage of Granzyme A^+^ CD8^+^ T cells suggesting further suppressive function of these cancer cell associated monocytes. In general, CD8^+^ T cells can play both effector and regulatory role in parasitic immunity (reviewed in[75]). CD8^+^ T cells mediated killing activities have been mostly directed and demonstrated against number of intracellular parasites that infect host cells[75]. How CD8^+^ T cells are regulating immunity against extracellular parasites is not fully understood. In filarial infections, CD8^+^ T cells exhibited a unique transcriptome in chronically-infected patients when compared to those with relatively acute infections, suggesting an importance of CD8^+^ cells in this infection[76]. Immune suppression in variety of helminth infections involves regulatory T cells[42]. For example, in onchocerciasis, Granzyme A/B expression was associated with Treg induction and subsequent immune suppression[77]. Induction of Treg were shown both in vitro[78,79] and in filarial-infected patients[80,81].

One important function of macrophages is their ability to phagocytose[82,83]. Macrophage phagocytosis plays a major role in tumor immune surveillance[84] in that antibody- dependent cellular phagocytosis mediated by macrophages contributes significantly to anti-tumor activity[85]. Macrophages that are polarized within the tumor microenvironment have increased phagocytic ability[86]. In the present study, exposure to MDA (Fig 4) and other cancer cell lines (S4 Fig) significantly induced the phagocytic ability of human monocytes to phagacytose bioparticles, suggesting that human monocytes exposed to cancer cell lines in vitro behave similarly to TAMs.

Our data suggest that despite the fact that helminth parasites and tumor cell lines are extraordinarily disparate, they share the ability to alter the phenotype of human monocytes although the nature of this alteration differed (see S1 Table). Nevertheless, similarities between the two types of stimuli in eliciting macrophage phenotypes similar to that of TAMs were observed, most notably in their ability to drive the surface expression of immune inhibitory molecules such as PDL1. Finally, utilizing a multidisciplinary approach to understand the mechanisms underlying immune evasion by both tumors and parasites could be beneficial to our understanding in both fields.

## Supporting information

S1 Table Similarities and differences between cancer cell lines and filarial parasites in shaping the phenotype and function of human monocytes. (TIFF)

S1 Fig Comparing media for monocyte cultures. Human monocytes were cultured in either complete DMEM media, complete RPMI media, or complete EMEM media for 48 hours. Cells were harvested, A) viability was measured using trypan blue exclusion, B) mRNA levels were measured by TaqMan real-time PCR and normalized to the levels of 18S rRNA, and C) surface expression PDL1 and CD206 was measured using flow cytometry. The data are expressed as the geometric mean (n = 2).
(TIFF)

S2 Fig mRNA expression of selected genes associated with inflammation, type 2, regulatory, and angiogenesis. Human monocytes were either unexposed (Mon) or exposed to CMFDA-labeled three different cancer cell lines (MDA, OVCAR, U87), or to live mf of *Brugia malayi* for 48 hours. CD45^+^/CMFDA^-^ monocytes were sorted and mRNA levels of selected genes associated with A) inflammation, B) type 2, C) regulatory and D) angiogenesis were measured by TaqMan real-time PCR and normalized to the levels of 18S rRNA. The data are expressed as the geometric mean with 95% confidence interval of 1/delta CT (n = 10). * *P*<0.05, ** *P*<0.005.
(TIFF)

S3 Fig Longer exposure of monocytes to mf results in increased PDL1 surface expression. Human monocytes were cultured in media alone or with live mf of *Brugia malayi* for 48 hours or 5 days. Cells were harvested and cell surface expression of PDL1 was measured using flow cytometry A) Flow histograms demonstrating cell surface expression in unexposed human monocytes and after exposure to mf, (isotype control, shaded areas; solid black lines, unexposed monocytes (Mon); and solid red lines, mf-exposed monocytes (Mon/mf), B) The frequency of PDL1^+^ cells and MFI of Mon and Mon/mf are shown. The data are expressed as the geometric mean (n = 2).
(TIFF)

S4 Fig Cancer cell lines induce the phagocytic ability of human monocytes. Human monocytes were cultured in media alone, or with CMFDA-labeled MDA, or live mf of *Brugia malayi* for 48hr. Cells were harvested and CD45^+^/CMFDA^-^ monocytes were sorted and cultured to measure phagocytosis of opsonized fluorescent- labeled *E*. coli bioparticles (see Materials and Methods). A) Flow histograms demonstrating percentage of phagocytic cells, B) Bars are shown as the percentage of phagocytic monocytes (FITC labeled *E*. *coli* positive).
(TIFF)

S5 Fig Cancer cell lines-exposed monocytes diminish CD4^+^ T and CD8^+^ T cells proliferation. Human monocytes were cultured in media alone, or with CMFDA-labeled-OVCAR, or CMFDA-labeled U87 for 48hr. Cells were harvested and CD45^+^/CMFDA^-^ monocytes were sorted and co-cultured with CFSE-labeled A) autologous or B) allogeneic lymphocytes in the presence of soluble anti-CD3 (10ug/ml) for an additional 4 days. Percent proliferation of CD4^+^ and CD8^+^ T cells was measured by flow cytometry either A and B) in the absence of antibody or C and D) in the presence of isotype control or anti-PDL1. The data are expressed as the geometric mean (n = 2).
(TIFF)

S6 Fig Longer exposure of monocytes to mf does not inhibit T cell proliferation. Human monocytes were cultured in media alone, or with live mf for 5 days. Cells were harvested and co-cultured with CFSE-labeled A) autologous or B) allogeneic lymphocytes in the presence of soluble anti-CD3 (10ug/ml) for an additional 4 days. Percent proliferation of CD4^+^ and CD8^+^ T cells was measured by flow cytometry either in the absence of antibody or in the presence of isotype control or anti-PDL1. The data are expressed as the geometric mean (n = 2).
(TIFF)

S7 Fig mRNA expression of selected genes associated with inflammation, type 2, regulatory, and angiogenesis following longer exposure. Human monocytes were either unexposed (Mon) or exposed to CMFDA-labeled three different cancer cell lines (MDA, OVCAR, U87), for either 5 or 7 days. CD45^+^/CMFDA^-^ monocytes were sorted and mRNA levels of selected genes associated with A) inflammation, B) type 2, C) regulatory, and D) angiogenesis were measured by TaqMan real-time PCR and normalized to the levels of 18S rRNA. The data are expressed as the geometric mean of 1/delta CT (n = 2).
(TIFF)

S8 Fig Exposure of monocytes to cancer cell line supernatants results in increased levels of PDL1 and CD206. Human monocytes were cultured in media alone or either with MDA, OVCAR, U87 cancer lines or supernatant from each cancer cell line for 24. Cells were harvested and cell surface expression PDL1 and CD206 was measured using flow cytometry (A) The frequency and B) MFI are shown. The data are expressed as geometric mean (n = 2).
(TIFF)

S1 Graphical Abstract Graphic to accompany the abstract. (PPTX)
