# Supplementary figures and images for: Similarities and differences between helminth parasites and cancer cell lines in shaping human monocytes: Insights into parallel mechanisms of immune evasion
Source: PLoS Negl Trop Dis. 2018 Apr 18;12(4):e0006404. doi: 10.1371/journal.pntd.0006404 (PMC5927465; doi:10.1371/journal.pntd.0006404)

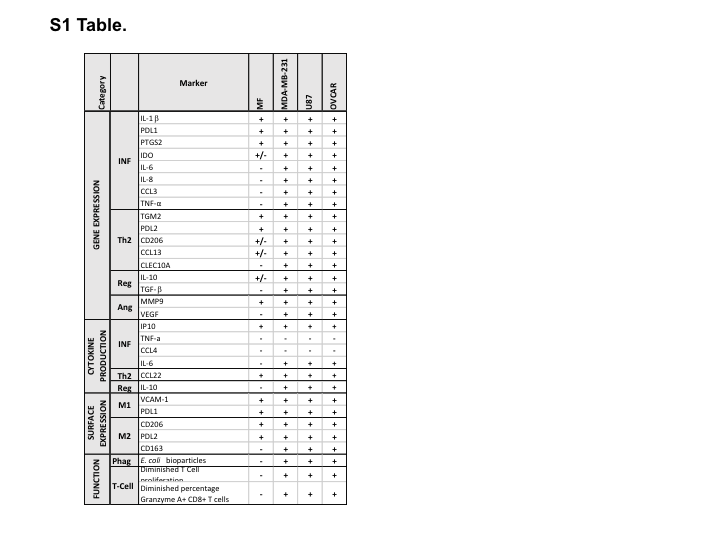

Supplement: S1 Table — (TIFF) [file pntd.0006404.s001.tiff]

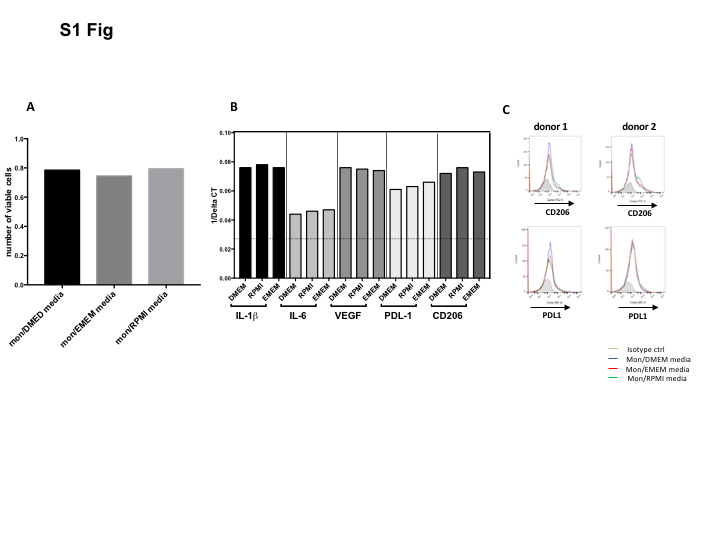

Supplement: S1 Fig — Human monocytes were cultured in either complete DMEM media, complete RPMI media, or complete EMEM media for 48 hours. Cells were harvested, A) viability was measured using trypan blue exclusion, B) mRNA levels were measured by TaqMan real-time PCR and normalized to the levels of 18S rRNA, and C) surface expression PDL1 and CD206 was measured using flow cytometry. The data are expressed as the geometric mean (n = 2). (TIFF) [file pntd.0006404.s002.tiff]

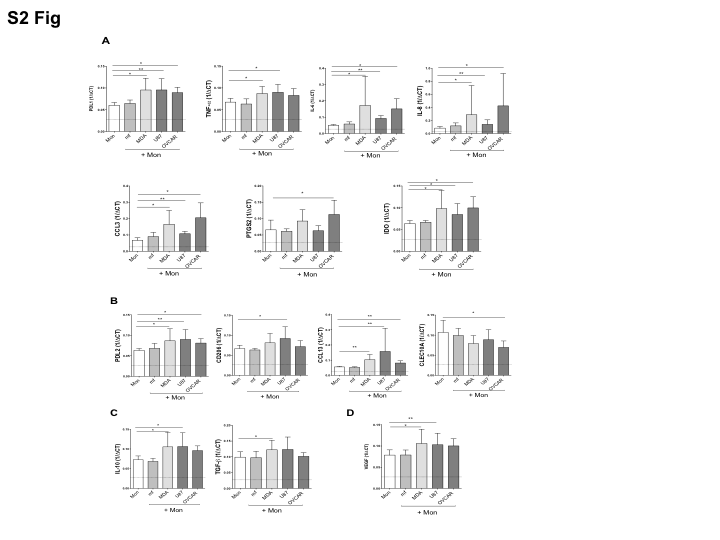

Supplement: S2 Fig — Human monocytes were either unexposed (Mon) or exposed to CMFDA-labeled three different cancer cell lines (MDA, OVCAR, U87), or to live mf of Brugia malayi for 48 hours. CD45+/CMFDA- monocytes were sorted and mRNA levels of selected genes associated with A) inflammation, B) type 2, C) regulatory and D) angiogenesis were measured by TaqMan real-time PCR and normalized to the levels of 18S rRNA. The data are expressed as the geometric mean with 95% confidence interval of 1/delta CT (n = 10). * P<0.05, ** P<0.005. (TIFF) [file pntd.0006404.s003.tiff]

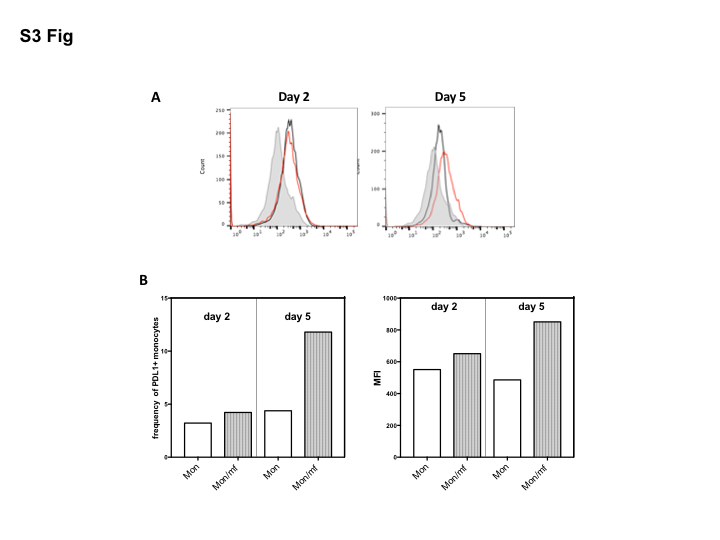

Supplement: S3 Fig — Human monocytes were cultured in media alone or with live mf of Brugia malayi for 48 hours or 5 days. Cells were harvested and cell surface expression of PDL1 was measured using flow cytometry A) Flow histograms demonstrating cell surface expression in unexposed human monocytes and after exposure to mf, (isotype control, shaded areas; solid black lines, unexposed monocytes (Mon); and solid red lines, mf-exposed monocytes (Mon/mf), B) The frequency of PDL1+ cells and MFI of Mon and Mon/mf are shown. The data are expressed as the geometric mean (n = 2). (TIFF) [file pntd.0006404.s004.tiff]

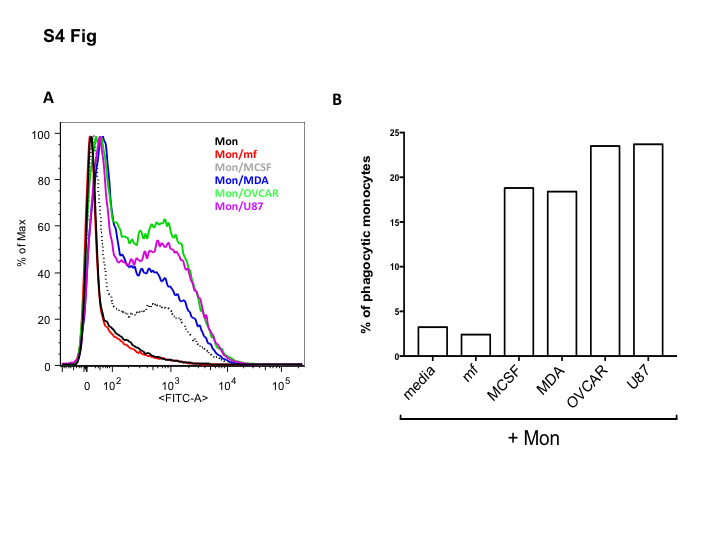

Supplement: S4 Fig — Human monocytes were cultured in media alone, or with CMFDA-labeled MDA, or live mf of Brugia malayi for 48hr. Cells were harvested and CD45+/CMFDA- monocytes were sorted and cultured to measure phagocytosis of opsonized fluorescent- labeled E. coli bioparticles (see Materials and Methods). A) Flow histograms demonstrating percentage of phagocytic cells, B) Bars are shown as the percentage of phagocytic monocytes (FITC labeled E. coli positive). (TIFF) [file pntd.0006404.s005.tiff]

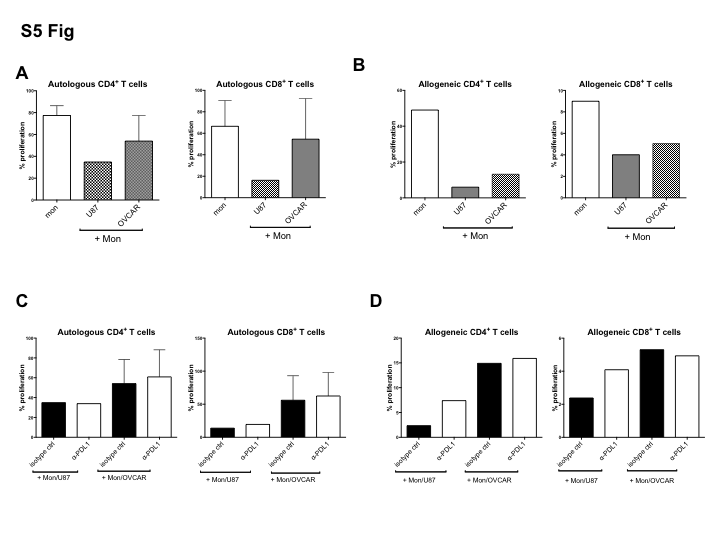

Supplement: S5 Fig — Human monocytes were cultured in media alone, or with CMFDA-labeled-OVCAR, or CMFDA-labeled U87 for 48hr. Cells were harvested and CD45+/CMFDA- monocytes were sorted and co-cultured with CFSE-labeled A) autologous or B) allogeneic lymphocytes in the presence of soluble anti-CD3 (10ug/ml) for an additional 4 days. Percent proliferation of CD4+ and CD8+ T cells was measured by flow cytometry either A and B) in the absence of antibody or C and D) in the presence of isotype control or anti-PDL1. The data are expressed as the geometric mean (n = 2). (TIFF) [file pntd.0006404.s006.tiff]

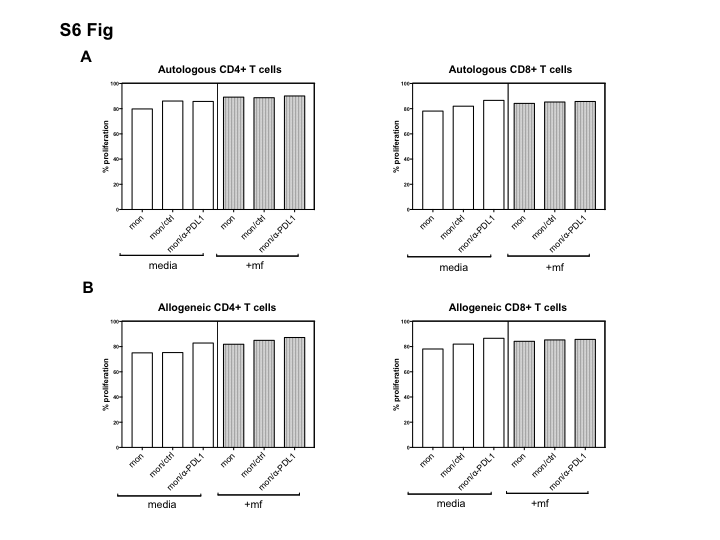

Supplement: S6 Fig — Human monocytes were cultured in media alone, or with live mf for 5 days. Cells were harvested and co-cultured with CFSE-labeled A) autologous or B) allogeneic lymphocytes in the presence of soluble anti-CD3 (10ug/ml) for an additional 4 days. Percent proliferation of CD4+ and CD8+ T cells was measured by flow cytometry either in the absence of antibody or in the presence of isotype control or anti-PDL1. The data are expressed as the geometric mean (n = 2). (TIFF) [file pntd.0006404.s007.tiff]

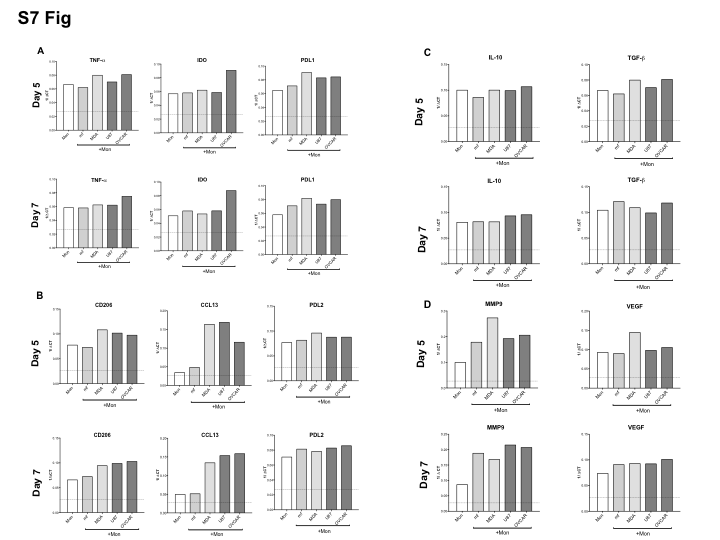

Supplement: S7 Fig — Human monocytes were either unexposed (Mon) or exposed to CMFDA-labeled three different cancer cell lines (MDA, OVCAR, U87), for either 5 or 7 days. CD45+/CMFDA- monocytes were sorted and mRNA levels of selected genes associated with A) inflammation, B) type 2, C) regulatory, and D) angiogenesis were measured by TaqMan real-time PCR and normalized to the levels of 18S rRNA. The data are expressed as the geometric mean of 1/delta CT (n = 2). (TIFF) [file pntd.0006404.s008.tiff]

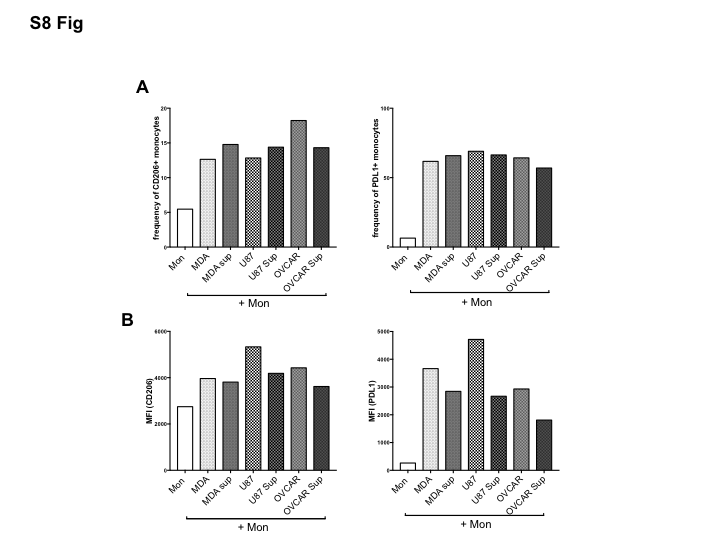

Supplement: S8 Fig — Human monocytes were cultured in media alone or either with MDA, OVCAR, U87 cancer lines or supernatant from each cancer cell line for 24. Cells were harvested and cell surface expression PDL1 and CD206 was measured using flow cytometry (A) The frequency and B) MFI are shown. The data are expressed as geometric mean (n = 2). (TIFF) [file pntd.0006404.s009.tiff]
