# Supplementary material for: Similarities and differences between helminth parasites and cancer cell lines in shaping human monocytes: Insights into parallel mechanisms of immune evasion
Source: PLoS Negl Trop Dis. 2018 Apr 18;12(4):e0006404. doi: 10.1371/journal.pntd.0006404 (PMC5927465; doi:10.1371/journal.pntd.0006404)
Supplement: S1 Graphical Abstract — (PPTX) [file pntd.0006404.s010.pptx]

## Slide 1
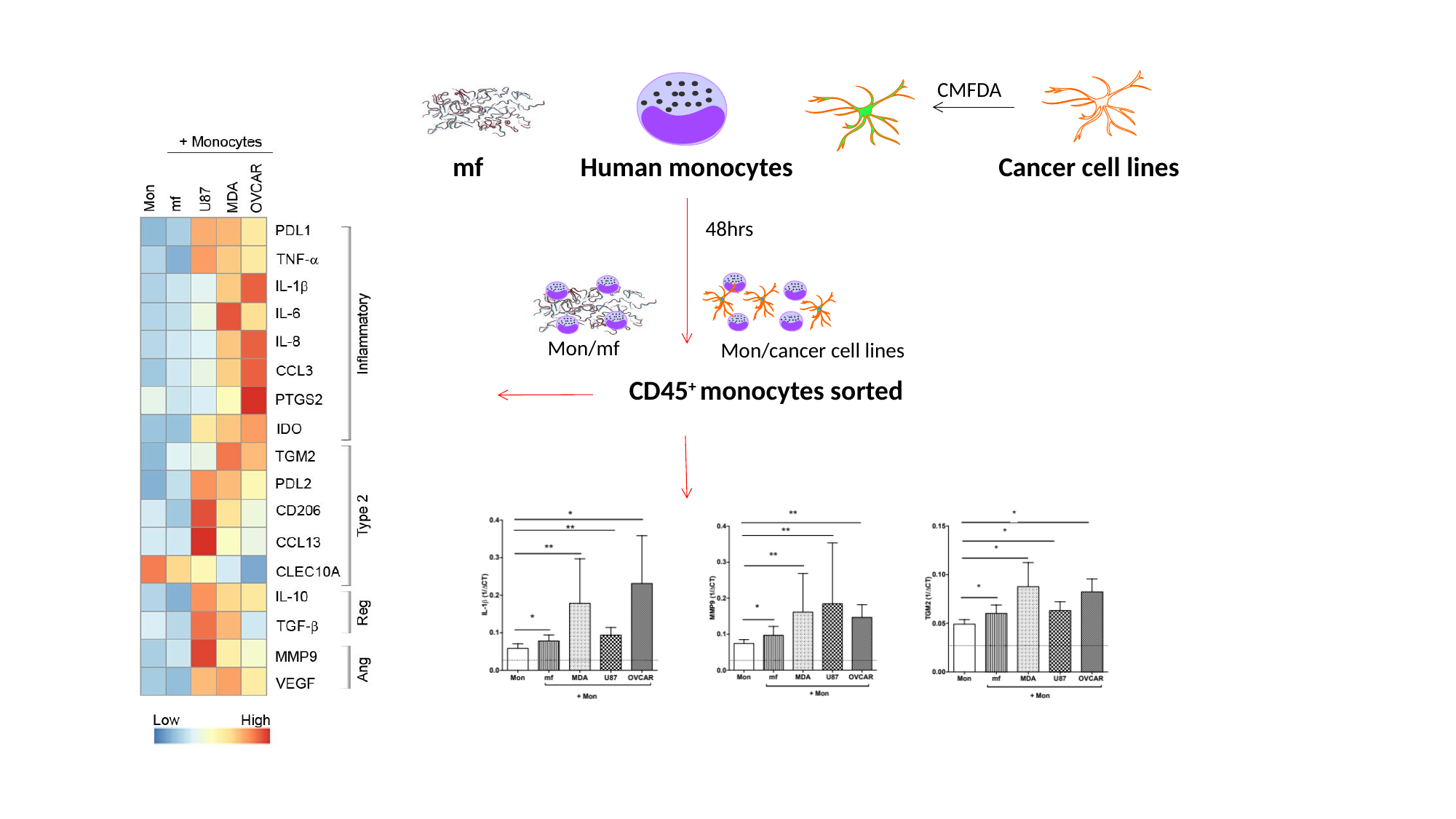

CMFDA
mf
Human monocytes
Cancer cell lines
48hrs
Mon/mf
Mon/cancer cell lines
 CD45+ monocytes sorted
